# Real‐world clinical outcomes of oligometastatic prostate cancer using SBRT: An Australian experience

**DOI:** 10.1002/bco2.70055

**Published:** 2025-10-01

**Authors:** Samantha Shekar, Megan Crumbaker, Anthony Joshua, Andrew Yam, Phillip Stricker, Carlo Yuen, David Ende, Benjamin Namdarian, James Thompson, Raji Kooner, Gordon O'Neill, Jeremy Mo, Hao‐Wen Sim, George Hruby, Farshad Kasraei, Annie Ho, Jeremy De Leon

**Affiliations:** ^1^ The Kinghorn Cancer Centre St Vincent's Hospital Darlinghurst NSW Australia; ^2^ Garvan Institute of Medical Research Darlinghurst NSW Australia; ^3^ St Vincent's Prostate Cancer Centre Darlinghurst NSW Australia; ^4^ St Vincent's Hospital Darlinghurst NSW Australia; ^5^ St Vincent's GenesisCare Darlinghurst NSW Australia; ^6^ University of Sydney Camperdown NSW Australia

**Keywords:** MDT, oligometastatic prostate cancer, prostate cancer, PSMA‐PET, SBRT

## Abstract

**Objectives:**

This work aimed to report real‐world outcomes from the use of SBRT to treat ADT naïve, PSMA‐detected oligometastatic prostate cancer (OPCa) and to assess disease and treatment characteristics in this heterogeneous population intersect or impact treatment response.

**Subjects and Methods:**

This retrospective single‐institution study examined PSMA‐PET–detected oligometastases (*n* = 1–5) in ADT‐naïve OPCa patients, treated with metastasis‐directed therapy (MDT) using SBRT delivered via MRI‐ or CT‐guided linear accelerator. Primary endpoint was biochemical progression free survival (PSA ≥ 25% if baseline <2 ng/mL or ≥2 ng/mL from nadir if baseline ≥ 2 ng/mL, start of systemic therapy, death). Secondary endpoints included time to PSA progression, time to next intervention, ADT‐free survival. Univariate and multivariate analyses were conducted for prognostic factors associated with bPFS, time to PSA progression and PSA50 response. Baseline clinical and treatment characteristics, PSA responses and local failure rates were analysed. Those with castrate‐resistant disease, prior systemic therapy or interval follow‐up of <6 weeks were excluded.

**Results:**

Sixty‐seven patients treated from January 2019 to August 2024 were analysed with a median follow‐up of 18.8 months. Ninety‐three oligometastatic lesions were treated; 55.3% were treated for nodal disease, 38.8% bone and 5% with lung disease. All lesions were PSMA‐detected with median SUVmax 6.3. Median bPFS was 22.1 m; TTNI was 28.8 m. Lower initial T stage and longer duration from OPCa diagnosis to MDT were associated with prolonged bPFS. Lower T stage and PSA doubling time >3 m at MDT were associated with prolonged time to PSA progression. Median PSA fall was 68.9%; PSA 50% response was observed in 55.2%. Twenty‐nine patients (43%) had a complete metabolic response after MDT. Median ADT‐free survival was not reached.

**Conclusions:**

MDT in PSMA‐PET‐detected OPCa can provide clinically meaningful disease control in a subset of patients. This study supports this approach but warrants continued prospective study and exploration into the castrate‐resistant setting.

## 1 INTRODUCTION

The oligometastatic hypothesis was first proposed by Hellman and Weichselbaum^1^ during the 1990s. It describes an intermediate state between localised primary disease and polymetastatic spread.^2^ In this state, malignant cells are proposed to have limited metastatic capacity, accompanied with less aggressive behaviour.^1^ In the current era, novel imaging techniques such as PSMA‐PET, with its high sensitivity and specificity, have improved disease characterisation of OPCa^3^ and enabled earlier disease detection. Use of MDT in this context is impacting established treatment paradigms of the timing of systemic therapy. Data from a recent meta‐analysis confirms that MDT offers a favourable toxicity profile and a chance to delay ADT initiation for at least 2 years in approximately half of cases.^4^

Currently in oligometastatic prostate cancer, prospective trials of SBRT demonstrate sustained periods of disease‐free survival, prolonging time to initiation of ADT^5^, ^6^, ^7^ and preventing the emergence of a castrate‐resistant state. The STOMP study comparing MDT with observation in OPCa, found ADT‐free survival of 21 months compared with 13 months, respectively.^5^ At 5 years, 34% of patients in the SBRT group remained ADT free. The median time to prostate‐specific antigen (PSA) progression was 10 months compared with 6 months in the surveillance group. QoL between the two groups remained comparable from baseline up to 1‐year follow‐up.^5^ Similar outcomes were reflected in a prospective trial of single treatment SBRT for bone and nodal oligometastases where 39% of patients had not distantly progressed and 48% remained ADT free at 2 years.^7^ Quality of life (QoL) measures were maintained with this strategy.^7^ Further to this, the ORIOLE trial demonstrated a 6‐month progression‐free survival (PFS) of 19% with observation compared with 61% in the MDT group.^8^ Median PFS with MDT was not reached compared with observation at 5.8 months.^8^ Of note, updated long‐term results from these trials did not reveal improvements in overall survival (OS), radiological PFS or castrate‐resistant prostate cancer (CRPC)‐free survival.^5^, ^8^

Despite few prospective large‐scale studies, current evidence supports improvement of ADT‐free survival, local control and progression‐free survival. This has significant implications in reducing the toxicity burden of systemic therapies especially cardiometabolic events, enabling treatment breaks in those with cumulative side effects and enhancing QoL.

At present, OPCa is defined based on the number of detectable lesions on conventional imaging. In an analysis of six original articles and 10 prospective trials, the majority limited the number of metastases included to up to five lesions with bone and lymph node metastases being commonly outlined when site‐specific criteria utilised.^9^ Although clinical parameters such as histology, time from cancer diagnosis to MDT or time from metastasis to MDT might serve to adjunct or supplement the standard definition.^10^, ^11^ The addition of these parameters to guide MDT may better reflect the heterogeneity of OPCa population, resulting in improved outcomes.^2^ Additionally, much of the data for MDT in OPCa identified metastatic disease with less sensitive imaging techniques rather than modern molecular imaging modalities.

We report real‐world data of PSMA‐PET detected, radiotherapy‐based MDT in OPCa to identify clinical characteristics associated with patient outcomes that may be used to refine patient selection for future MDT‐trials.

## 2 SUBJECTS AND METHODS

### 2.1 Study design and participants

This is a retrospective study conducted at St Vincent's Hospital (Darlinghurst, Sydney, Australia). The study was approved by St Vincent's Hospital Human Research Ethics Committee (2021/ETH11891). The study population included patients with oligometastatic prostate cancer treated with stereotactic doses either delivered either via an MRI guided daily adapted platform or conventional linac between 1st January 2019 to 1st August 2024.

Men with histologically confirmed prostate cancer, PSMA‐PET/CT imaging consistent with oligometastatic disease (defined as fewer than five lesions) and considered appropriate for MDT with SBRT to metastatic lesions were included. All patients had previous definitive management of their primary prostate tumour with surgery or radiotherapy (salvage or adjuvant radiotherapy to the prostate bed or pelvis allowed) prior to MDT. Standard dosing and fractionation for SBRT of specific oligometastatic lesions were adopted. Patients could have received adjuvant ADT during definitive management or salvage treatment. A minimum follow‐up period of 6 weeks after each MDT course was required. Patients were typically reviewed every 3 to 6 months after MDT with repeat PSA and imaging repeated at 6‐ to‐12‐month intervals or earlier based on symptoms or change in PSA kinetics.

Patients with castration‐resistance or that had received systemic therapy (including chemotherapy, androgen receptor pathway inhibitor or continuous androgen deprivation therapy) prior to oligometastatic treatment were excluded. Individuals without a minimum of 6 weeks' follow‐up data (PSA or imaging) were excluded.

### 2.2 Study measures

Clinical data were obtained including age and PSA at prostate cancer (PC) diagnosis, Gleason score, primary tumour/nodal status/metastatic disease classification, treatment of primary tumour, use of ADT prior to MDT, SUVmax of treated lesions and the number and location of oligometastases. PSA kinetics including PSA at time of MDT and doubling time prior to, and post MDT were collected. Post‐treatment clinical data were obtained for site of recurrence and choice of treatment at subsequent distant progression.

The primary outcome was biochemical progression free survival (bPFS) including events of interests such as PSA failure, start of systemic therapy (i.e., ADT) or death by any cause. As per The Prostate Cancer Trials Working Group 3 (PCWG3),^11^ PSA failure/progression was defined as a PSA rise of ≥25% and ≥2 ng/mL from nadir (if baseline PSA was ≥2 ng/mL) or PSA rise of ≥ 25% from nadir (if baseline PSA was <2 ng/mL at MDT initiation).^12^ Indications to start ADT included symptomatic progression, progression to more than five metastases or local progression of baseline detected metastases. Patients who did not start ADT or have PSA failure at the time of last follow‐up were censored at that timepoint.

Secondary outcomes were time to PSA progression, time to next intervention defined as a change in therapy after SBRT (including repeat SBRT to oligometastases), PSA30/50/90 responses (PSA reduction of ≥30%, ≥50% and ≥90% from baseline following treatment), maximum PSA fall post MDT, PSA responses at 3/6/12 months, ADT‐free survival and local or distant failure rates.

### 2.3 Statistical analysis

Demographic and clinical data were summarised using descriptive statistics for categorical and continuous variables. Survival analysis using the Kaplan–Meier method was conducted for median biochemical PFS (bPFS), time to next intervention and time to PSA progression. Time to event outcomes were censored if the event was not met or end of follow‐up was reached, whichever came first. Univariable Cox regression analysis was conducted to identify variables associated with bPFS, time to PSA progression and PSA50 response. Significant variables found to be associated on univariable analysis (*p* < 0.05) were included in a multivariable analysis. Patients that received ADT peri‐MDT were excluded from univariate and multivariate analysis. Descriptive statistics were used to analyse the maximum fall in PSA levels, PSA response rates and rates of local progression. All statistical analysis was undertaken with R.

## 3 RESULTS

### 3.1 Patient, disease and treatment characteristics

Sixty‐seven patients were identified and analysed. Table 1 summarises the baseline characteristics of the population. The median age was 75 years. Median initial PSA was 6.8 ng/mL (range, 1.4–34 ng/mL). Fifty patients (76.9%) had a Gleason score of 6 to 7 while 15 patients (23.1%) with Gleason 8 to 9 score. Most patients had a primary tumour classification of T2 (51.6%) followed by T3a/3b (48.4%). Initial treatment of the prostate primary was largely radical prostatectomy alone (31.3%) or radical prostatectomy with salvage radiotherapy (46.3%). Most patients (77.6%) did not receive ADT peri‐treatment of the primary. Peri‐treatment ADT use was defined as either concurrently with definitive RT or neoadjuvant/adjuvant to radical prostatectomy in node positive settings. Those that did receive ADT were primarily in the adjuvant setting (11.9%) with testosterone recovery post cessation in the majority (93.3%). One patient remained castrate post ADT cessation.

**TABLE 1:** Baseline patient and disease characteristics.

Characteristic (*n* = 67)	Value
Age (year) at treatment, median (range)	75 (60–89)
Initial PSA at diagnosis (ng/mL), median (range)	6.8 (1.4–34)
Gleason score (%)	
6	2 (3.1)
7	48 (73.8)
8–9	15 (23.1)
Primary tumour classification (%)	
T2	33 (51.6)
T3a/3b	31 (48.4)
Node status at initial diagnosis (%)	
N0	31 (48.4)
N1	9 (14.1)
Nx/cN0	24 (37.5)
Type of treatment at PC diagnosis (%)	
Radical prostatectomy	21 (31.3)
Radical prostatectomy/salvage radiotherapy	31 (46.3)
Radical prostatectomy/adjuvant radiotherapy	7 (10.4)
Definitive radiotherapy/ADT	3 (4.5)
Definitive radiotherapy	3 (4.5)
Brachytherapy	2 (3)
Salvage prostatectomy	2 (3)
ADT during primary treatment (%)	
Yes	15 (22.4)
No	52 (77.6)
Indication for ADT commencement at primary treatment	
Adjuvant	8 (11.9)
Concurrent with salvage radiotherapy	4 (6)
Concurrent with definitive radiotherapy	3 (4.5)
Testosterone recovery post ADT at primary treatment (%)	
Yes	14 (93.3)
Unknown	1 (6.7)

Abbreviations: ADT, androgen deprivation therapy; PC, prostate cancer; PSA, prostate‐specific antigen.

Baseline treatment characteristics are reported in Table 2. Sixty‐five patients (97%) received MDT for metachronous disease and two patients (3%) treated for synchronous disease. Time between diagnosis and first MDT was 85.1 months (1.7–266.6 months). Patients had mostly one (73.1%) or two (19.4%) oligometastases. The treated lesions were pelvic nodes (47.8%), bone (38.8%), abdominal nodes (7.5%) and lung (6%). Thirty‐nine patients (58.2%) were treated with SBRT on conventional LINAC and 28 patients (41.8%) were treated with MRI‐guided SBRT on MR‐LINAC. All patients were staged with enhanced imaging including PSMA‐PET/CT (95.5%) ± Bombesin scan (4.5%). Post‐SBRT follow‐up imaging was with enhanced modality (53.7%) or conventional imaging (6%) with a large proportion (40.3%) with no follow‐up imaging post treatment as was not clinically indicated.

**TABLE 2:** Baseline treatment characteristics.

Characteristic (*n* = 67)	Value
Time between diagnosis and MDT (month), median (range)	85.1 (1.7–266.6)
Timing of MDT treatment (%)	
Synchronous	2 (3)
Metachronous	65 (97)
Number of metastases (%)	
1	49 (73.1)
2	13 (19.4)
3	2 (3)
4	3 (4.5)
Location metastases (%)	
M0	32 (47.8)
M1a	5 (7.5)
M1b	26 (38.8)
M1c	4 (6)
Treatment site (%)	
Pelvic lymph node	32 (47.8)
Abdominal lymph node	5 (7.5)
Bone	26 (38.8)
Lung	4 (6)
Type of radiotherapy (%)	
SBRT	39 (58.2)
MRI‐Linac	28 (41.8)
Staging imaging (%)	
PSMA‐PET or Bombesin	67 (100)
Conventional	0
Follow‐up imaging	
Enhanced	36 (53.7)
Conventional	4 (6)

Abbreviations: ADT, androgen deprivation therapy; MDT, metastases‐directed therapy, MRI, magnetic resonance imaging; PC, prostate cancer; PSA, prostate‐specific antigen; PSMA‐PET, prostate‐specific membrane antigen positron emission tomography; RT, radiotherapy; SBRT, stereotactic radiotherapy.

### 3.2 PSA kinetics and response

**FIGURE 1:**
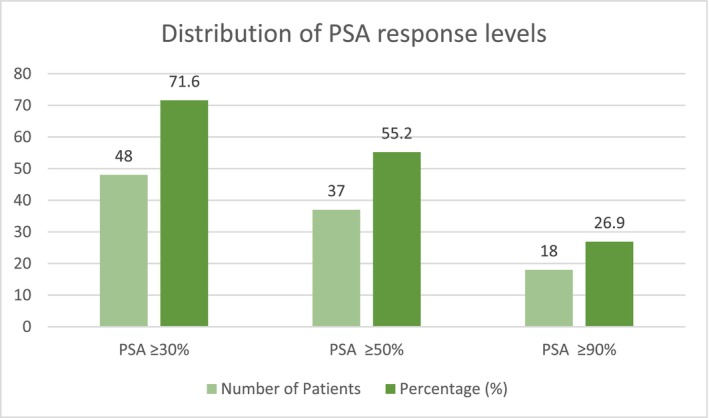
. Summary of PSA response levels.

Median PSA prior to the first MDT was 0.59 ng/mL (0.03–10 ng/mL) with most PSA values ranging between 0.2 and 2.0 ng/mL (59.7%). Most patients (95.5%) had a PSA doubling time prior to MDT of greater than 3 months. This remained consistent post treatment with majority of patients (94%) still having a PSA doubling time of greater than 3 months. We found 60 patients (89.6%) had either PSA decline or stability post MDT. Of these 60 patients, all demonstrated a PSA response at 3 months, 48 (80%) at 6 months and 30 (50%) at 12 months. Forty‐eight patients (71.6%) achieved a PSA reduction of at least 30%, 37 patients (55.2%) of 50% and 18 patients (26.9%) of 90% or greater from baseline (Figure 1). The median maximum PSA fall was 68.9%, mean maximum PSA fall was 61.3% with the largest observed PSA fall of 99.8%. On univariate analysis of 37 patients with a PSA50 response, a significant association (*p* = 0.03) was found with a longer duration from PCa diagnosis to MDT initiation with a median of 8.6 years.

### 3.3 Clinical outcomes

**FIGURE 2:**
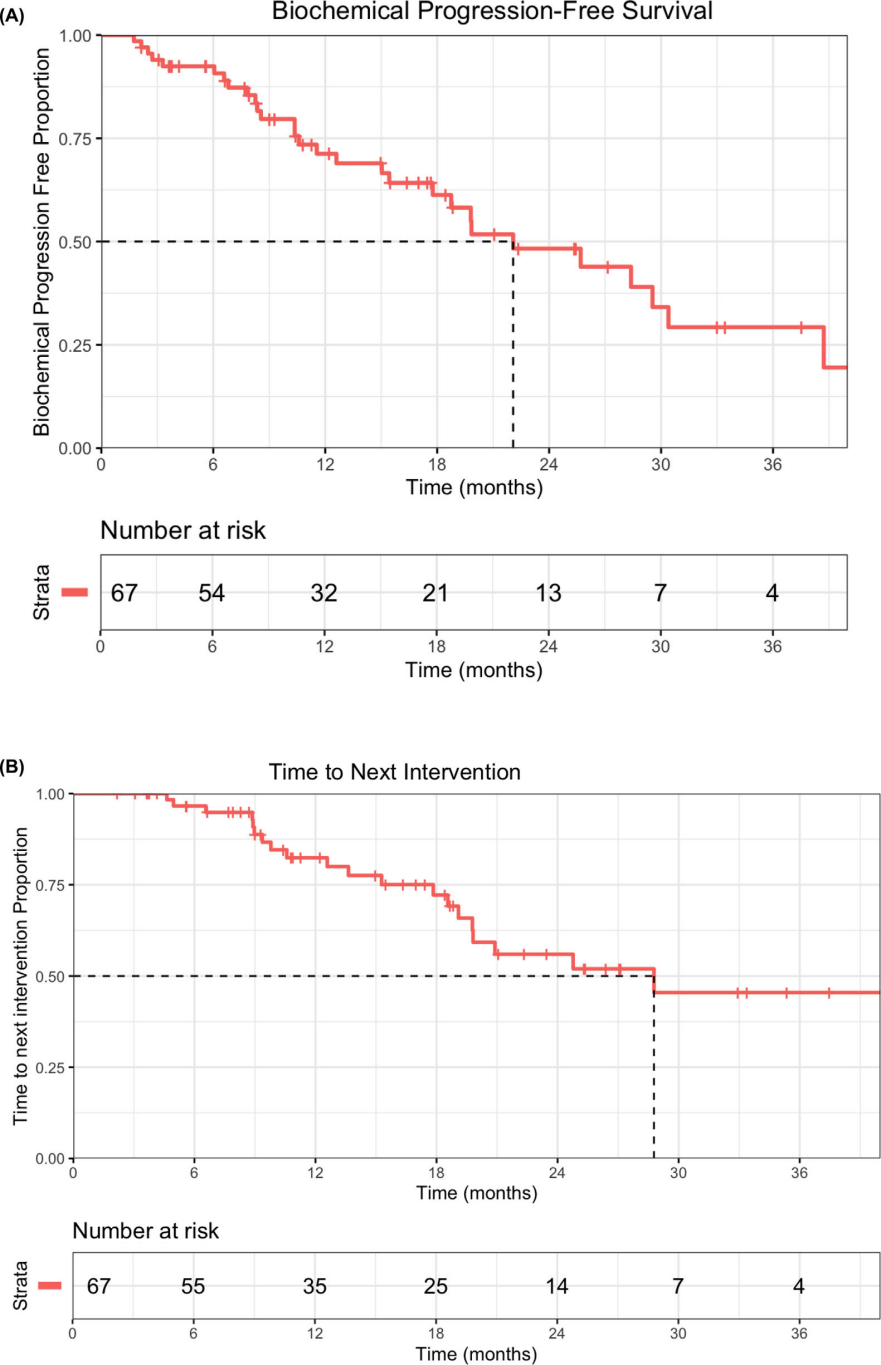
(A) Biochemical progression‐free survival after radiotherapy. (B) Time to next intervention after radiotherapy.

**FIGURE 3:**
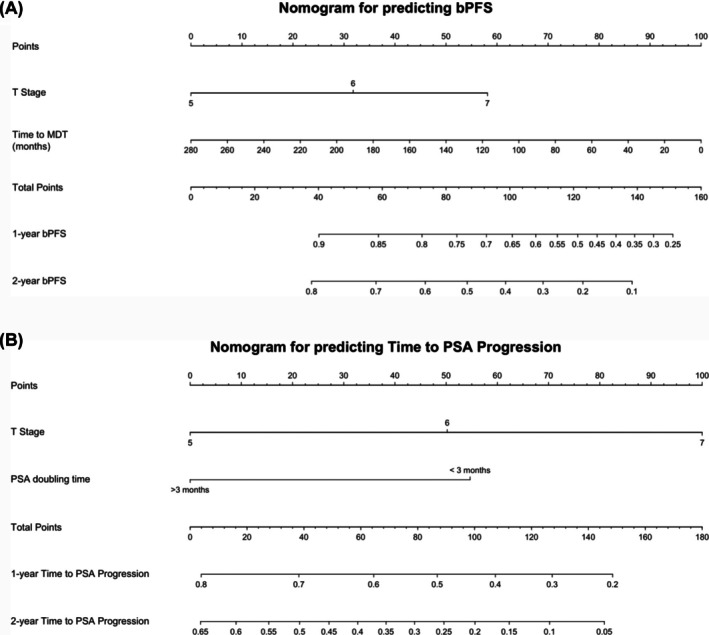
(A) Nomogram for predicting bPFS. (B) Nomogram for predicting time to PSA progression.

Median follow‐up time was 18.8 months (range, 16.4–25.4 months). Median bPFS was 22.1 months (95% confidence interval [CI], 17.8 months—not reached; Figure 2A). On univariate analysis (*n* = 67), lower initial T stage and longer duration from PCa diagnosis to MDT were associated with improved bPFS (Table 3). On multivariate analysis, initial T stage was statistically significant (*p* = 0.02) with a hazard ratio of 2.47 (95% CI 1.15–5.34) suggesting a higher T stage was associated with an increased risk of biochemical progression (Table 3). Time from PCa diagnosis to MDT was marginally significant (*p* = 0.06) with a hazard ratio of 0.99. Median time to PSA progression was 25.7 months (95% confidence interval [CI], 17.7 months—not reached). On multivariate analysis (*n* = 28), lower T stage and PSA doubling time greater than 3 months prior to MDT were associated with prolonged time to PSA progression (Table 3). Nomograms predicting 1‐ and 2‐year bPFS and time to PSA progression based on predictive factors included in the multivariate analysis are summarised in Figure 3.

**TABLE 3:** Univariate and multivariate analyses of clinical factors.

Univariate analysis for factors associated with bPFS, PSA50 response and time to PSA progression		
Characteristic	HR (95% CI)	*p* value
**bPFS**		
Age at diagnosis of oligometastatic disease	0.96(0.91–1.01)	0.13
T stage		0.02
T3	2.48(1.14–5.37)	
T2	0.4(0.19–0.87)	
Node status		0.22
N1	1.75(0.7–4.38)	
Nx/N0	0.57(0.23–1.43)	
M stage		0.49
M1	1.31(0.6–2.84)	
M0	0.76(0.35–1.65)	
Gleason score		0.26
8–9	1.52(0.72–3.19)	
6–7	0.66(0.31–1.38)	
Location of treated oligometastases		
Node	0.57(0.27–1.23)	0.15
Bone	1.75(0.82–3.74)	0.15
Lung	0.28(0.04–2.18)	0.23
PSA doubling time prior to MDT		0.49
>3 months	0.6(0.14–2.61)	
<3 months	1.66(0.38–7.18)	
Baseline PSA at MDT initiation	0.97(0.81–1.16)	0.80
Time from diagnosis to MDT initiation	0.99(0.98–1.00)	0.03
**PSA50 response**		
Age at diagnosis of oligometastatic disease	1.03(0.98–1.08)	0.25
T stage		0.14
T3	0.56(0.26–1.21)	
T2	1.78(0.82–3.83)	
Node status		0.07
N1	0.19(0.02–1.39)	
Nx/N0	5.39(0.72–40.41)	
M stage		0.84
M1	1.22(0.16–9.13)	
M0	0.82(0.11–6.15)	
Gleason score		0.87
8–9	0.94(0.48–1.84)	
6–7	1.06(0.54–2.07)	
Location of treated oligometastases		
Node	0.59(0.29–1.21)	0.15
Bone	1.7(0.83–3.5)	0.15
Lung	0.51(0.12–2.25)	0.38
PSA doubling time prior to MDT		0.87
>3 months	1.19(0.16–8.84)	
<3 months	0.84(0.11–6.22)	
Baseline PSA at MDT initiation	0.99(0.85–1.16)	0.93
Time from diagnosis to MDT initiation	0.99(0.985–0.999)	0.03
**Time to PSA progression**		
Age at diagnosis of oligometastatic disease	0.99(0.93–1.05)	0.68
T stage		0.02
T3	2.59(1.13–5.95)	
T2	0.39(0.17–0.89)	
Node status		0.96
N1	0.97(0.39–0.95)	
Nx/N0	1.03(0.41–2.59)	
M stage		0.93
M1	0.91(0.12–6.82)	
M0	1.1(0.15–8.33)	
Gleason score		0.89
8–9	1.05(0.49–2.26)	
6–7	0.95(0.44–2.04)	
Location of treated oligometastases		
Node	0.54(0.24–1.2)	0.13
Bone	1.86(0.83–4.19)	0.13
Lung	0.88(0.11–6.92)	0.90
PSA doubling time prior to MDT		< 0.001
>3 months	0.05(0.01–0.34)	
<3 months	21.21(2.94–153.94)	
Baseline PSA at MDT initiation	0.92(0.76–1.11)	0.39
Time from diagnosis to MDT initiation	0.997(0.989–1.00)	0.56

Multivariate analysis for factors associated with bPFS and time to PSA progression		
Characteristic	HR (95% CI)	*p* value
**bPFS**		
T stage	2.47(1.15–5.34)	0.02
Time to MDT	0.99(0.98–1.00)	0.06
**Time to PSA progression**		
T stage	2.38(1.02–5.57)	0.04
PSA doubling time prior to MDT	0.06(0.009–0.48)	0.007

Abbreviations: bPFS, biochemical progression‐free survival; CI, confidence interval; HR, hazard ratio; MDT, metastasis directed therapy; PSA, prostate specific antigen.

Ninety‐three lesions were detected on PSMA‐PET and subsequently treated. The median SUVmax of treated lesions was 6.3 (2.5–59.2). Of patients that had follow‐up imaging post MDT, 29 (43%) patients had a complete metabolic response. The 26 patients (39%) that did not have follow‐up imaging, was due to a lack of clinical indication for repeat imaging. Progression of treated oligometastases on follow‐up imaging occurred in 10 patients (15%). Distant progressive disease occurred in 15 patients (22%). Of these patients nine (60%) recurred with bone metastases, 5 (33%) with lung lesions and 1 (7%) in nonregional nodes.

The median time to next intervention was 28.8 months with (95% confidence interval [CI], 19.8 months—not reached; Figure 2B). Twenty‐two patients required subsequent treatment. Treatment modalities at next intervention included SBRT in 11 patients (50%), systemic therapy in seven patients (31.8%) followed by combined SBRT/ADT in four patients (18.2%).The median ADT‐free survival was not reached, with 11 patients starting on palliative ADT by the data cut‐off date.

## 4 DISCUSSION

Our study describes a real‐world experience treating OPCa with SBRT at a median follow‐up of 18.8 months. In our cohort of 67 patients, median bPFS and TTNI after SBRT were 22.1 months and 28.8 months, respectively. Time to PSA progression was 25.7 months. The median maximum PSA fall was 68.9%. PSA30/50/90 responses were observed in 71.6%, 55.2% and 26.9% of patients, respectively. The median ADT‐free survival was not reached. We performed an exploratory analysis to see if a particular subgroup did well or may have been unsuitable for MDT. On multivariate analysis, a lower initial T stage was significantly associated with prolonged bPFS. Lower initial T stage and a PSA doubling time greater than 3 months prior to MDT were predictive of prolonged time to PSA progression.

Management of OPCa with MDT remains an investigational area,^13^ and it is yet to be determined whether patients should be managed with observation, systemic therapy, metastasis‐directed therapy (MDT) or a combination of both.^6^ Real‐world evidence with large cohorts supporting the rationale of MDT in OPCa is lacking. At present, prospective data in support of MDT has been established through the Surveillance or Metastasis‐Directed Therapy for Oligometastatic Prostate Cancer Recurrence (STOMP) trial.^5^ It demonstrated treatment of oligometastases primarily via SBRT, significantly improved ADT free survival to 21 months compared with 13 months with surveillance alone.^5^ Early phase trials have combined MDT with short‐term standard systemic therapy in OPCa.^14^, ^15^ This therapeutic strategy leverages the noninvasive advantage of SBRT with the synergy of hormone therapy to enhance radiation benefit, limiting the medical castration duration.^14^ The EXTEND trial found MDT with intermittent hormone therapy improved PFS compared with hormone therapy alone (not reached vs. 15.8 months, respectively).^14^ Furthermore, 24‐month toxicity data reported in the PEACE V‐STORM trial highlighted that elective nodal pelvic radiotherapy (ENRT) and MDT with 6 months of ADT for mostly PSMA‐PET detected oligorecurrent nodal prostate cancer had acceptable toxicity rates with no clinically meaningful difference in acute gastrointestinal or genitourinary toxicity or QoL subdomains compared with baseline.^15^ Patient reported outcomes were comparable between the groups, a promising indicator for MDT safety and utility.

Additionally, a plethora of retrospective series have suggested that SBRT in OPCa provides optimal metastasis control is well tolerated and beneficial in deferring time to hormonal therapy and prolonging biochemical PFS.^2^, ^16^, ^17^, ^18^, ^19^ Our study contributes to the growing literature, providing an overview of the Australian experience in this space.

This study represents the perspective of real‐world experience in a large single institution using both standard and MRI‐Linac guided radiotherapy techniques to treat oligometastatic lesions. We included 67 patients in the ADT naive setting, with a similar sample size to current retrospective data and prospective trials reporting on populations on the order of 50 to 60 patients. With consideration to events such as PSA failure, initiation of systemic therapy or death by any cause, this study demonstrated a favourable median bPFS response in keeping with current prospective evidence.^5^, ^6^, ^8^ With regard to known prognostic clinical factors, our findings indicate patients with low volume OPCa (1–2 metastases) treated in the metachronous setting with a slow peri‐RT PSA doubling time, lower initial T stage and with a longer duration of time from OPCa diagnosis to MDT were the optimal population subset for intervention with MDT.

With the advancement in imaging and availability of 68Ga prostate‐specific membrane antigen (PSMA) PET, there are higher rates of early detection of micro‐metastatic disease and improved patient selection for MDT compared with conventional imaging.^20^ In the oligometastatic recurrent state, 78% of panellists of The Advanced Prostate Cancer Consensus Conference voted for next‐generation imaging methods to restage biochemically recurrent PC.^17^ Prospective evidence suggests PSMA‐PET results are highly predictive of 3‐year freedom from progression in patients undergoing SBRT for biochemical recurrence after radical prostatectomy, highlighting the valuable predictive potential of this imaging modality.^21^ All patients in our study were staged via enhanced imaging modalities with the majority (88%) having undergone prior radical prostatectomy. The use of enhanced staging may also aid in stratifying cohorts with higher subclinical disease burden than expected where systemic therapy alone is best suited^2^ versus detection of disease at lower thresholds where MDT may be of most benefit. Follow‐up staging in our study population varied, likely owing to accessibility of continued PSMA‐PET surveillance. Thirty‐six (54%) patients had follow‐up PSMA‐PET scans while 4 (6%) had conventional imaging. Of note, the SUVmax of an oligometastatic lesion did not significantly prolong bPFS, time to PSA progression or achievement of PSA50 response.

The findings in this series were comparable to well cited trials including STOMP^5^ and ORIOLE.^8^ In our study, time to next intervention was 28.8 months and included 11 patients who received a subsequent MDT course. We found this comparable to a similar US retrospective study^2^ and median time to ADT seen in STOMP.^5^ As seen with previous reports, patterns of progression were observed primarily with bone and nodal recurrence, noted equally (11.9%) in our population.^18^ Varying locoregional treatment approaches are currently being adopted and electing to use ADT concomitantly remains an open question. The early phase POPSTAR study of single‐fraction SBRT for bone and nodal oligometastases met its endpoints of feasibility and tolerability.^7^ Much like STOMP, nearly half patients treated with SBRT were ADT free at 24 months.^7^ A strength of our study, distinct to ORIOLE, STOMP and POPSTAR, was the use of PSMA‐PET imaging in detection of oligometastases, aligning with the currently shifting use of enhanced imaging modality from conventional standards for early detection and more rigorous selection of patients undergoing treatment for OPCa. Furthermore, there is small cohort preliminary data supporting the feasibility of the MRI‐Linac technique in OPCa.^16^ Approximately 42% of our population underwent treatment with MRI‐Linac guidance, adding to the limited data available for this treatment.

In the context of Australian experience, our data aligns with both prospective and retrospective literature.^6^, ^19^ In these studies, the majority of patients (~88%) had undergone prior radical prostatectomy and had PSMA‐PET detected oligometastatic spread to nodal and osseous sites, reflective of our study.^6^, ^19^ Kneebone et al. demonstrated high rates of local control with no in‐field failures on repeat PSMA‐PET for all patients with biochemical failure post MDT.^6^ Despite the variability in follow‐imaging, our data confirms 43% of all included patients had a complete metabolic response at treated sites on repeat PSMA‐PET. Similar to prospective evidence, predictive factors such as time from diagnosis to SBRT, Gleason score, baseline PSA and location of oligometastases did not appear to predict improved bPFS.^6^ However, an exploratory analysis of our data did confirm lower initial T stage was associated with a prolonged bPFS and time to PSA progression, in conjunction with a slower PSA doubling time >3 months prior to MDT. In a similar retrospective series, complete PSA response was seen in 27% with a median bPFS of 1.1 years comparable to results of our analysis.^19^

While the results are promising, this study has several limitations. It was retrospective with a small sample size. Seven individuals without follow‐up data (PSA or imaging) were excluded from analysis despite receiving oligometastatic treatment. Clinical selection bias may have evolved over time, in those participants that were offered whole pelvic RT and concurrent ADT versus an MDT approach for nodal disease. This is reflective in the median time from definitive treatment to SBRT of 7 years. In regard to ADT‐free survival, there were not enough events to perform a robust statistical analysis and hence the event rate remains inconclusive. Furthermore, ADT free survival remains an inconsistent endpoint as ADT initiation may be influenced by patient and clinician preference based on PSA doubling time. The retrospective nature inherently opens to biases and may affect the statistical power of the data. Although patients were treated at a single centre, care was across multiple clinicians thus impacting timing of imaging, PSA testing and PSA threshold for ADT administration. Owing to accessibility of enhanced imaging, follow‐up staging was inconsistent in this cohort impacting the interpretation of local progression of baseline‐detected oligometastatic lesions. Castrate‐resistant patients were not included in this study but further exploration in this setting is certainly warranted and is currently being investigated through the STAMPEDE2 trial of SBRT compared with Lu‐PSMA‐617 treatment in metastatic prostate cancer.^22^

## 5 CONCLUSION

Both standard and MRI‐Linac guided MDT in PSMA‐PET detected OPCa can be effective in prolonging biochemical PFS, time to next intervention and PSA progression across a heterogeneous population of varying prostate cancer subtypes. The data from this study supports this approach but warrants continued prospective study and exploration into the castrate resistant setting.

## AUTHOR CONTRIBUTIONS

S.S. wrote the manuscript alongside M.C. A.J., J.D.L., S.S., J.M. and H.‐W.S. performed statistical analysis. All other authors reviewed the manuscript and provided editorial suggestions.

## CONFLICT OF INTEREST STATEMENT

The authors declare their affiliations with St Vincent's Hospital Sydney, The Kinghorn Cancer Centre Sydney, St Vincent's Clinic Sydney, St Vincent's Prostate Cancer Centre Sydney and St Vincent's GenesisCare NSW. The authors have no other conflicts of interest to disclose in relation to this manuscript.
